# T2-prepared segmented 3D-gradient-echo for fast T2-weighted high-resolution three-dimensional imaging of the carotid artery wall at 3T: a feasibility study

**DOI:** 10.1186/s12938-016-0276-9

**Published:** 2016-12-28

**Authors:** Jian Zhu, Axel Bornstedt, Nico Merkle, Naifeng Liu, Wolfgang Rottbauer, Genshan Ma, Volker Rasche

**Affiliations:** 1 Department of Cardiology, Zhongda Hospital, Southeast University, Nanjing, China; 2 Department of Internal Medicine II, University Hospital of Ulm, Ulm, Germany

**Keywords:** Vessel wall imaging, Carotid artery, T2-preparation, Gradient echo

## Abstract

**Background:**

The multi-contrast assessment of the carotid artery wall has become an important diagnostic tool for the characterization of atherosclerotic plaque and vessel wall thickening. For providing the required T1-, T2-, and proton density weighted contrast, multi-slice turbo spin echo (TSE) techniques are normally applied. The straightforward extension of the TSE techniques to volumetric imaging of large sections of the carotid arteries is limited by the resulting long acquisition times. Where the acquisition of a T1-weighted contrast can be accelerated by applying a T1-weighted fast gradient echo technique, acceleration of the T2-weighted contrast is not as straightforward.

**Methods:**

In this work, the combination of a T2 preparation and a conventional fast gradient echo technique (T2P-3DGE) was evaluated for rapid acquisition of a T2-weighted image contrast. Acquisition parameters were optimized in an initial in vitro study in direct comparison to the conventional T2-weighted TSE (T2W-3DTSE) technique. Subsequently, the T2P-3DGE technique was evaluated in vivo.

**Results:**

In direct comparison, the T2P-3DGE sequence provided similar T2 contrast as the respective T2W-3DTSE sequence. After correction of an observed intensity offset, most likely caused by the additional T1-weighting of the T2P-3DGE sequence, no significant difference between the two T2-weighted sequences were observed in phantom data. The good correlation of the image contrast between the two sequences was confirmed in the initial in-vivo study, proving a potential reduction of the scan time for T2P-3DGE to 25% of the respective T2W-3DTSE technique.

**Conclusion:**

The in vitro as well as the in vivo results clearly indicate the potential of the T2P-3DGE technique for providing similar T2 image contrast as in the conventional techniques. Thereby, the acquisition times could be substantially reduced to about 25% of the respective 3D-TSE technique.

## Background

Stroke is one of the leading causes of morbidity and mortality worldwide. Atherosclerotic carotid artery narrowing is currently a relevant clinical risk factor for developing stroke [1]. However, the degree of luminal stenosis alone is a relatively poor predictor of subsequent neurological events [2–5]. During recent years, the concept of vulnerable atherosclerotic lesions has evolved as potentially improved predictor for neurological events [4, 5]. Due to its versatile contrast properties and potentially high spatial resolution, magnetic resonance imaging (MRI) has great potential to provide high-fidelity images of the vessel wall, thus enabling visualization and quantification of the plaque morphology, identification of tissue types, and the detection of other pathological features associated with vulnerability [6–8].

Most commonly, plaque imaging has been achieved by combining a double-inversion recovery black-blood preparation [9] with two-dimensional multi-slice (MS2D) data acquisition by means of turbo spin echo (TSE) techniques [6, 7, 10–12]. Proper adjustment of the TSE sequence parameters allows multi-contrast MRI including T1 (T1W), T2 (T2W) and proton density (PDW) weighted image contrast. Since high-spatial resolution is required and the T2 relaxation constants of the different plaque components are rather short ranging from 10.4 to 112.4 ms [13], the maximal length of the readout duration (and hence the maximal possible TSE acceleration factor k) has to be limited to avoid image blurring caused by severe T2 apodization. Without compromising spatial resolution, this technique allows for the acquisition of a single slice in the minute time range using reasonably long echo trains.

For volume coverage, most studies so far have combined TSE imaging with multi-slice 2D acquisitions to measure the required volume-of-interest (VOI). A major limitation of these approaches results from their non-isotropic spatial resolution and the rather long acquisition times. The straight extension of the TSE techniques to three-dimensional (3D) coverage of large sections of the arteries of interest [14] has been limited by the resulting even longer acquisition times. For shortening of the acquisition time, previous investigators have applied combined gradient and spin echo [15] (GRASE), reduced field of view [16, 17], fast gradient echo [18], and 3D-TSE with variable flip angle [19] (VFA-SPACE) techniques. Where the gradient echo based approaches do not provide sufficient T2 contrast but short acquisition times, the VFA-SPACE technique proved almost similar T2 contrast as the conventional T2W-TSE techniques but may show some longitudinal magnetization recovery over the rather long echo train and still requires rather long acquisition times. It has been shown earlier that by the application of a T2 preparation sequence [20], T2 image contrast can also be obtained in combination with fast gradient echo techniques [21].

It is the objective of this study to investigate the potential of T2-prepared three-dimensional spoiled gradient echo (T2P-3DGE) techniques for providing T2 contrast in reasonable acquisition time.

## Theory

The application of a T2-preparation module (T2P) is well known from MRI coronary angiography for improving the contrast between blood and e.g. the myocardium [20, 22]. Assuming complete longitudinal relaxation between subsequent T2 preparation pulses, the resulting signal intensities $S\left( {T_{1} ,T_{2} ,T_{2}^{*} } \right)$ can be approximated by the signal intensity $S_{GE} \left( {T_{1} ,T_{2}^{*} } \right)$ resulting from a conventional spoiled gradient echo technique multiplied by the additional T2 weighting of the magnetization introduced by the T2 preparation, yielding:

$$ S\left( {T_{1} ,T_{2} ,T_{2}^{*} } \right) = M_{0} \frac{{\sin \left( \alpha \right)\;\left( {1 - e^{{ - {{T_{R} } \mathord{\left/ {\vphantom {{T_{R} } {T_{1} }}} \right. \kern-0pt} {T_{1} }}}} } \right)}}{{\left( {1 - \cos \left( \alpha \right)\;e^{{ - {{T_{R} } \mathord{\left/ {\vphantom {{T_{R} } {T_{1} }}} \right. \kern-0pt} {T_{1} }}}} } \right)}}e^{{ - \frac{{T_{E} }}{{T_{{_{2} }}^{ * } }}}} e^{{ - \frac{{T_{P} }}{{T_{2} }}}} = S_{GE} \left( {T_{1} ,T_{2}^{*} } \right)\;e^{{ - \frac{{T_{P} }}{{T_{2} }}}} $$

with *T*_*P*_ being the T2P preparation time, and α the excitation flip angle. By choosing a sufficiently short *T*_*E*_, *T*_2_* effects can be almost neglected and the resulting signal is governed by the *T*_*2*_ decay of the tissue superimposed by some additional *T*_*1*_ relaxation.

**Fig. 1:**
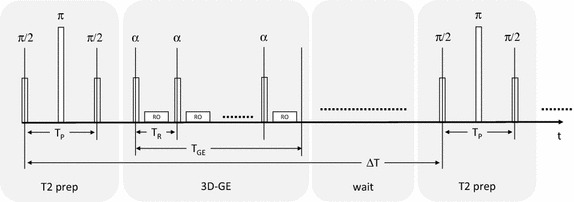
Schematically representation of the T2P-3DGE sequence. For simplification, only the RF pulses are provided. The readout phase including phase-encoding and readout gradients is shown as a single block RO. *T*_*P*_ T2 preparation time; *T*_*GE*_ gradient echo readout block; *∆T* interval between subsequent T2 preparations

A schematic diagram of the T2P-3DGE sequence is provided in Fig. 1. In principle, the sequence comprises a conventional three-dimensional spoiled steady-state gradient echo sequence. Prior to the acquisition of each segment (3D-GE), a T2 preparation module (T2 prep) is played out to ensure T2-weighted magnetization preparation. To enable recovery of the magnetization, after the acquisition of each segment a waiting period (wait) is interwoven. For ensuring black-blood contrast, motion-sensitizing gradients [23] were applied before and after the refocusing pulse of the T2-prep module (not shown).

## Methods and materials

All imaging was performed on a 3T whole-body system (Achieva, Philips Healthcare, Best, The Netherlands) equipped with a high-performance gradient system capable of a maximum gradient amplitude of 40 mT m^−1^ and maximum slew rate of 200 T m^−1^ s^−1^.

### In vitro evaluation

For optimization of the T2-prepared fast gradient echo sequence (T2P-3DGE), an agarose gel phantom containing several insets with different concentrations of iron oxide particles was used. Depending on the iron concentration, the resulting T2 values ranged from roughly 18.9 to 96.3 ms as mapped by a conventional multi-spin echo technique with subsequent fitting of a mono-exponential T2 decay curve to the multi-echo data. The optimization of the acquisition parameters included shot interval ∆T (the interval between subsequent application of the T2 preparation), the preparation time T_P_, the acquisition duration T_GE_ (number of gradient echoes per shot times T_R_) and the excitation flip angle α. All data were acquired with an 8-element head coil. Coil sensitivity patterns and receiver gain settings were corrected before image analysis. The resulting image intensities of a certain T2P-3DGE parameter set were quantitatively compared to the T2W-3DTSE reference scan (Table 1: T2W-3DTSE). The optimization target was reaching similar image contrast by the T2P-3DGE sequence as provided by the T2W-3DTSE technique.

**Table 1:** Acquisition parameters (TR: repetition time; TE: echo time; ΔTE: echo spacing; α: flip angle; FOV: field-of-view; DIR: double inversion recovery; MS: motion sensitized; SPIR: spectral presaturation with inversion recovery)

	Turbo spin echo (TSE)			Gradient echo (GE)	
	T1W-3DTSE	PDW-3DTSE	T2W-3DTSE	3DGE	T2P-3DGE
TR (ms)	952	1905	2250	6.4	6.4
TE (ms)	11	10	52	3.3	3.3
ΔTE (ms)	12	10.4	10.4	NA	NA
α [deg]	90	90	90	20	20
FOV (mm) RL/AP/FH	150 × 200 × 20	150 × 200 × 20	150 × 200 × 25	150 × 200 × 25	150 × 200 × 25
Slice thickness/gap (mm)	2.0/−1.0	2.0/−1.0	2.0/−1.0	2.0/−1.0	2.0/−1.0
Resolution (mm) RL/AP/FH	0.45 × 0.45 × 2	0.45 × 0.45 × 2	0.45 × 0.45 × 2	0.45 × 0.45 × 2	0.45 × 0.45 × 2
k-Space filling pattern	Centric	Centric	Linear	Linear	Linear
Black-blood	DIR	DIR	DIR	MS	MS
Fat saturation	SPIR	SPIR	SPIR	SPIR	SPIR
NSA	1	1	1	3	3
Turbo factor	11	12	9	20	20
SENSE factor	1	2	2	1	1
Halfscan factor	0.675	0.675	0.675	1	1
Scan time	6 min 14 s	7 min 16 s	7 min 41 s	6 min 36 s	6 min 36 s

### In vivo evaluation

After optimization of the T2P-3DGE acquisition parameters, the proposed technique was evaluated in 10 patients [male/female 9/1, mean age 66 ± 6 years (59–73)], scheduled for a conventional MRI investigation of the carotid artery wall. The MR protocol was approved by the Ethic Committee of University Hospital of Ulm with signed written informed consent obtained from all patients before data acquisition.

### In vivo imaging protocol

The comprehensive MRI investigation included a fast survey, followed by a coil sensitivity map for homogenization of the images and for parallel imaging reconstruction. An additional inflow angiogram was acquired for accurate planning. Vessel wall images were obtained by three-dimensional TSE (3DTSE) techniques with proton density (PDW-3DTSE), T1 (T1W-3DTSE), and T2 (T2W-3DTSE) image contrast and a fast three-dimensional gradient echo technique with (T2P-3DGE) and without (3DGE) T2 preparation. Black-blood image contrast was obtained by double inversion recovery (3D-TSE) or a driven-equilibrium motion-sensitized preparation (3DGE, [23]). For T2P-3DGE, the motion sensitizing gradients were combined with the T2 preparation module. The order of the sequences was randomized for each patient. A detailed description of the imaging parameters is provided in Table 1. All images were acquired in axial orientation centered at the bulbus of the carotid bifurcation. For suppression of swallowing motion artifacts, a pencil beam navigator [23] positioned at the epiglottis was applied before the respective sequence block in all measurements.

All data were acquired utilizing a dedicated two segment four-element carotid coil (Philips Research Europe, Germany). Either segment comprises two independent coil elements with spatial extent of 65 × 50 mm^2^ each.

### Data analysis

All patient data was transferred to a medical workstation (ViewForum, Philips Healthcare, Best, The Netherlands). For SNR measurements, a region-of-interest (ROI) was manually placed in the vessel wall and the vessel lumen, and the SNR was calculated as the fraction of the mean signal intensity and the standard deviation of the respective ROI. The *CNR* was calculated as $CNR\left( {a,b} \right) = \frac{{\left| {S_{a} - S_{b} } \right|}}{{0.5\left( {\sigma_{a} + \sigma_{b} } \right)}}$, with *S* being the signal intensity and σ being the respective standard deviation. The outer and inner vessel areas were calculated according to manually drawn contours, and the vessel wall area calculated as the respective difference. All analyses were performed in a slice located in the ACC, identified manually 10 mm caudal to the bulbus.

A nonparametric two-tailed Mann–Whitney U test was performed for assessment of the significance of the results. P values below 0.05 were considered significant. All statistical analyses were performed with the real statistics package for Excel (http://www.real-statistics.com).

## Results

### In vitro

**Fig. 2:**
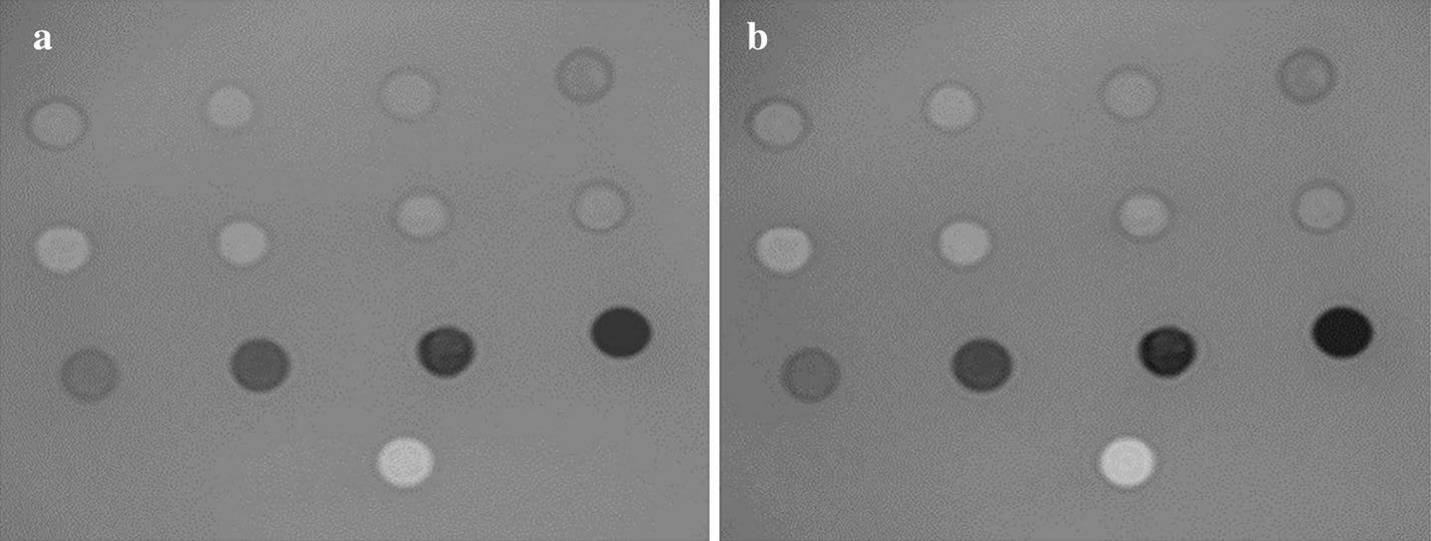
Appearance of the agarose phantom acquired with the conventional T2W-3DTSE (**a**) and the proposed T2PW-GE (**b**) technique

**Fig. 3:**
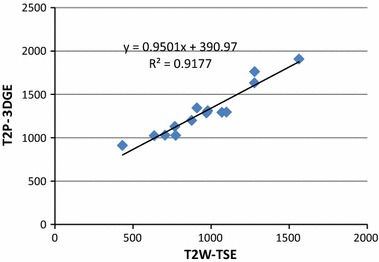
Resulting signal intensities for different T2 species in the phantom

The in vitro study revealed that choosing the T2P-3DGE sequence parameters as: ∆T = 1000 ms, T_GE_ = 128.4 ms, T_P_ = 50 ms, and α = 20° provided very similar T2 weighting of the resulting image as the conventional T2W-3DTSE sequence (Fig. 2). Quantitative comparison (Fig. 3) revealed an excellent correlation (R^2^ = 0.92) of the T2W-3DTSE and T2P-3DGE image intensities. However, an offset, most likely caused by the additional T1 weighting of the T2P-3DGE sequence, can be appreciated. Furthermore, a slight increase of the intensity differences can be observed for the T2P-3DGE approach [I_T2W-TSE_ = 0.95 (I_T2P-3DGE_ – I_T2P-Offset_)]. After correction for the offset no significant (p = 0.72) differences between the two approaches were observed.

### In vivo

The imaging protocol could be completed in all patients. One patient had to be excluded due to severe motion artifacts in all images. Acquisition time per slice for the T2W-3DTSE technique resulted to 23 s. Respective acquisition time for the T2P-3DGE approach resulted to 5.4 s. To match signal to noise in both techniques, three signal averages were used in most patients for the T2P-3DGE approach yielding a realistic acquisition time per slice of 16.2 s. Navigator efficiency resulted higher than 95% in all cases and respective prolongation of the acquisition time could be neglected.

**Fig. 4:**
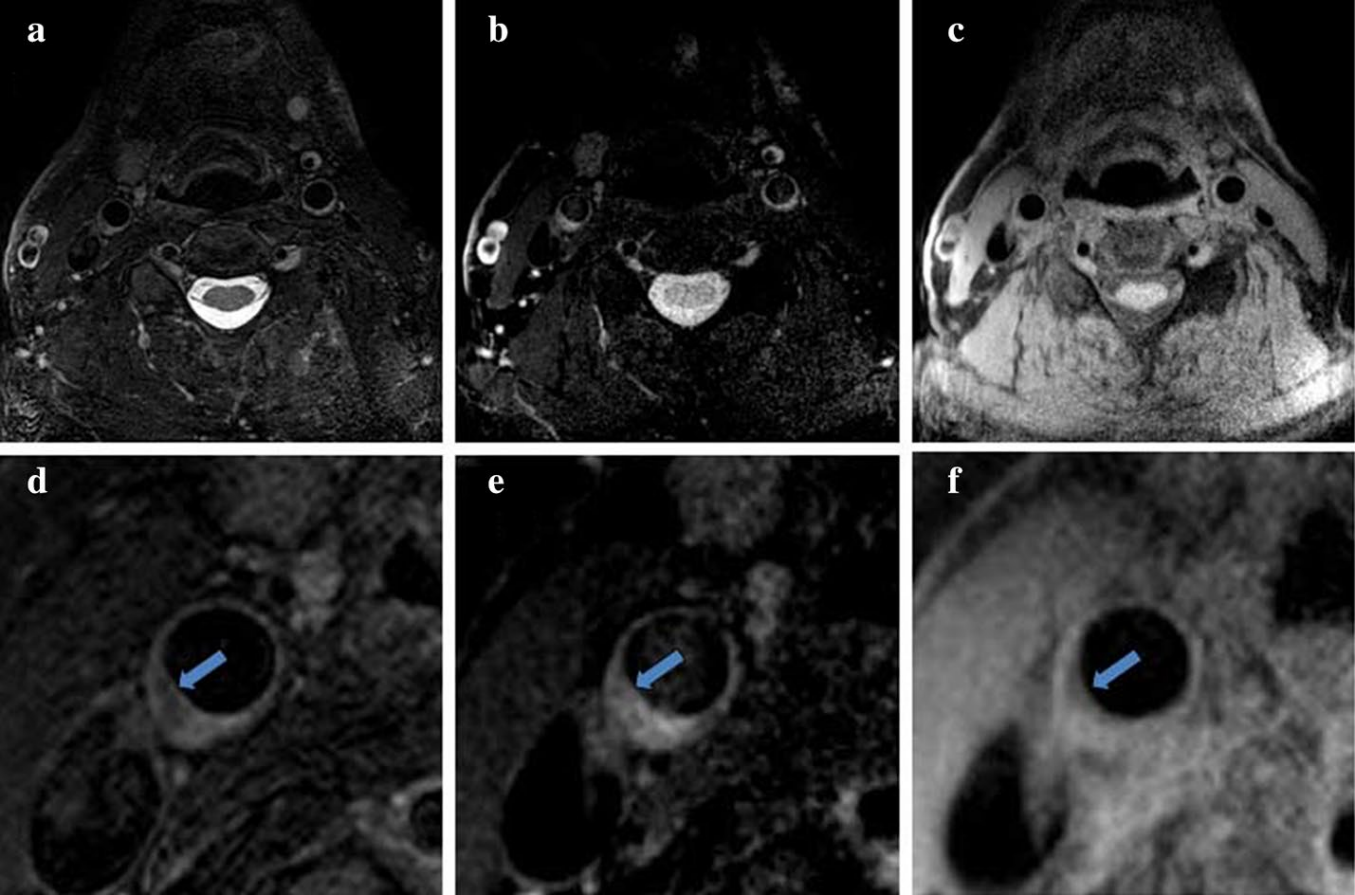
Overview images (*top*) and close-ups images (*bottom*) acquired by T2W-3DTSE (**a**, **d**), T2P-3DGE (**b**, **e**) and 3DGE (**c**, **f**)

**Fig. 5:**
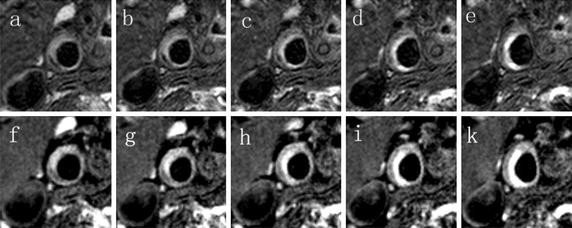
Consecutive slices of an atheroma acquired with the T2W-3DTSE (**a**-**e**) and the T2P-3DGE (**f**-**k**) technique

Qualitative comparison of the 3DGE technique (Fig. 4c) with the T2P-3DGE technique (Fig. 4b) clearly revealed the increased T2 weighted contrast in the T2P-3DGE data. In direct comparison with the respective T2W-3DTSE image (Fig. 4a), a very similar contrast can be appreciated in the T2P-3DGE image. Obvious differences appeared in areas of short T1 components (e.g. the myelon), where the additional T1 weighting substantially contributed to the final image intensity. Close-ups of the lesion (Fig. 4d–f) support the T2W contrast of the T2P-3DGE technique. Figure 5 shows five consecutive slices of a substantially enlarged vessel wall acquired with the T2W-3DTSE (Fig. 5a–e) in direct comparison with the T2P-3DGE (Fig. 5f–k) technique. A very similar contrast in the lesion can be appreciated by both techniques.

For the T2 prepared gradient echo technique, a significant (p < 0.05) increase in SNR for the vessel wall and lumen, and a clear trend (p < 0.06) to improved wall-lumen CNR were observed (see Table 2).

**Table 2:** Quantitative analysis of the SNR and CNR properties of the T2-prepared gradient echo technique and the conventional T2-weighted turbo spin echo technique

	T2W-3DTSE		T2P-3DGE		p
	Mean	σ	Mean	σ	
*SNR* (wall)	6.84	1.61	12.92	7.05	0.01
*SNR* (lumen)	1.24	0.17	2.46	0.75	0.007
*CNR* (wall, lumen)	1.82	0.33	2.74	1.12	0.06

**Fig. 6:**
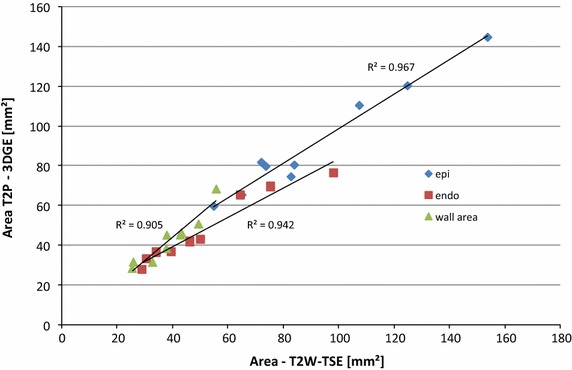
Comparison of the areas of the vessel wall obtained from the endo- and epi-vascular contours and the respective vessel wall area acquired by the conventional T2W-TSE technique (*x-axis*) and the proposed T2P-3DGE technique (*y-axis*)

No significant differences and good correlation (see Fig. 6) were observed between the two techniques for the inner (p = 0.06) and outer vessel wall (p = 0.64) areas, with a trend to underestimation of the inner area, causing a significant (p = 0.024) overestimation of the resulting vessel wall area (see Table 3).

**Table 3:** Quantitative analysis of the resulting geometrical properties of the vessel derived by the T2-prepared gradient echo technique and the conventional T2-weighted turbo spin echo technique

	T2W-3DTSE		T2P-3DGE		p
	Mean	σ	Mean	σ	
*A*_*endo*_ (mm^2^)	90.1	31	90.1	28	0.06
*A*_*epi*_ (mm^2^)	51.1	23.3	48	17.7	0.64
*A*_*wall*_ (mm^2^)	39	10	42	12	0.024

*A*_*endo*_ area of the vessel lumen; *A*_*epi*_ area of the vessel including lumen and wall; *A*_*wall*_ area of the vessel wall

## Discussion

T2 preparation (T2P) is commonly used in coronary magnetic resonance angiography (MRA) to improve the CNR between blood (long T2) and myocardium (short T2), hence improving vessel conspicuity. Further application of T2P includes the rapid generation of images with multiple T2 weightings for T2 mapping of the myocardium. In this study, T2P was combined with rapid three-dimensional gradient echo imaging (T2P-3DGE) for providing T2 contrast in carotid artery wall imaging.

The study shows that by proper adjustment of the acquisition parameters, the T2P-3DGE provides similar contrast as the conventional T2-weighted turbo spin echo (T2W-3DTSE) technique. Differences result from the additional T1 weighting of the T2P-3DGE sequence, which cause signal enhancement especially from short T1 species like fat. A slight underestimation of the vessel wall area is observed. This may be attributed to the superior *CNR* in the T2P-3DGE technique, which provides a better delineation of the vessel wall. Whether these contrast differences and slight differences in the vessel wall area will cause diagnostic consequences must be proven by further clinical evaluation.

The main motivation of applying the T2P-3DGE sequence is the shortening of acquisition time in lengthy 3D acquisitions. In our case, the acquisition time per slice could theoretically be reduced to 25% without obvious changes in the resulting lesion contrast. However, due to the intrinsic lower SNR in T2P-3DGE, in our current setup three signal averages are required for ensuring sufficient SNR, whereas in the T2W-3DTSE a single signal average appears sufficient. Thus currently, the acquisition time gain is 25%, only. The currently superior *SNR* and *CNR* in the T2P-3DGE techniques show the potential for further reduction of the measurement time. In combination with high field-strength or improved coil designs, the related increased SNR may even enable full utilization of the theoretical gain in acquisition speed. But even for the current setup, the application of the T2P-3DGE technique in combination with multiple signal averages appears promising for increasing the volume coverage by a factor of three without any prolongation of the overall acquisition time. Furthermore, when keeping the covered volume constant, the required signal averages can be applied for further reduction of motion artifacts caused by swallowing or pulsatile motion of the arteries.

The TSE-based as well as GE-based sequences tested here, have been optimized regarding providing sufficient SNR while maintaining scan times in reasonable limits. Potential further reduction of acquisition time may rise from using variable flip angle TSE approaches enabling longer echo trains and by exploiting parallel imaging techniques for T2P-3DGE.

## Conclusions

The T2-prepared gradient echo approach can be applied for providing similar T2-weighted contrast as known from the conventional T2W-TSE imaging technique in shorter image acquisition time. The impact of the slight contrast variations introduced by the intrinsic T1 weighting of the 3DGE sequence on the diagnostic accuracy has to be evaluated in further clinical studies.

## References

[1] Sacco RL, Wolf PA, Gorelick PB (1999). Risk factors and their management for stroke prevention: outlook for 1999 and beyond. Neurology.

[2] National Institute of Neurological Disorders and Stroke Stroke and Trauma Division, North American Symptomatic Carotid Endarterectomy Trial (NASCET) investigators (1991). Clinical alert: benefit of carotid endarterectomy for patients with high-grade stenosis of the internal carotid artery. Stroke.

[3] European Carotid Surgery Trialists’ Collaborative Group (1991). MRC European carotid surgery trial: interim results for symptomatic patients with severe (70–99%) or with mild (0–29%) carotid stenosis. Lancet.

[4] Naghavi M, Libby P, Falk E, Casscells SW, Litovsky S, Rumberger J, Badimon JJ, Stefanadis C, Moreno P, Pasterkamp G (2003). From vulnerable plaque to vulnerable patient: a call for new definitions and risk assessment strategies: part I. Circulation.

[5] Naghavi M, Libby P, Falk E, Casscells SW, Litovsky S, Rumberger J, Badimon JJ, Stefanadis C, Moreno P, Pasterkamp G, Fayad Z, Stone PH, Waxman S, Raggi P, Madjid M, Zarrabi A, Burke A, Yuan C, Fitzgerald PJ, Siscovick DS, de Korte CL, Aikawa M, Airaksinen KE, Assmann G, Becker CR, Chesebro JH, Farb A, Galis ZS, Jackson C, Jang IK, Koenig W, Lodder RA, March K, Demirovic J, Navab M, Priori SG, Rekhter MD, Bahr R, Grundy SM, Mehran R, Colombo A, Boerwinkle E, Ballantyne C, Insull W, Schwartz RS, Vogel R, Serruys PW, Hansson GK, Faxon DP, Kaul S, Drexler H, Greenland P, Muller JE, Virmani R, Ridker PM, Zipes DP, Shah PK, Willerson JT (2003). From vulnerable plaque to vulnerable patient: a call for new definitions and risk assessment strategies: part II. Circulation.

[6] Yuan C, Mitsumori LM, Beach KW, Maravilla KR (2001). Carotid atherosclerotic plaque: noninvasive MR characterization and identification of vulnerable lesions. Radiology.

[7] Saam T, Hatsukami TS, Takaya N, Chu B, Underhill H, Kerwin WS, Cai J, Ferguson MS, Yuan C (2007). The vulnerable, or high-risk, atherosclerotic plaque: noninvasive MR imaging for characterization and assessment. Radiology.

[8] Fabiano S, Mancino S, Stefanini M, Chiocchi M, Mauriello A, Spagnoli LG, Simonetti G (2008). High-resolution multicontrast-weighted MR imaging from human carotid endarterectomy specimens to assess carotid plaque components. Eur Radiol.

[9] Edelman RR, Chien D, Kim D (1991). Fast selective black blood MR imaging. Radiology.

[10] Serfaty JM, Chaabane L, Tabib A, Chevallier JM, Briguet A, Douek PC (2001). Atherosclerotic plaques: classification and characterization with T2-weighted high-spatial-resolution MR imaging—an in vitro study. Radiology.

[11] Fayad ZA, Fuster V (2000). Characterization of atherosclerotic plaques by magnetic resonance imaging. Ann N Y Acad Sci.

[12] Cai J, Hatsukami TS, Ferguson MS, Kerwin WS, Saam T, Chu B, Takaya N, Polissar NL, Yuan C (2005). In vivo quantitative measurement of intact fibrous cap and lipid-rich necrotic core size in atherosclerotic carotid plaque: comparison of high-resolution, contrast-enhanced magnetic resonance imaging and histology. Circulation.

[13] Sharma R (2002). MR imaging in carotid artery atherosclerosis plaque characterization. Magn Reson Med Sci.

[14] Crowe LA, Gatehouse P, Yang GZ, Mohiaddin RH, Varghese A, Charrier C, Keegan J, Firmin DN (2003). Volume-selective 3D turbo spin echo imaging for vascular wall imaging and distensibility measurement. J Magn Reson Imaging.

[15] Luk-Pat GT, Gold GE, Olcott EW, Hu BS, Nishimura DG (1999). High-resolution three-dimensional in vivo imaging of atherosclerotic plaque. Magn Reson Med.

[16] Bornstedt A, Bernhardt P, Hombach V, Kamenz J, Spiess J, Subgang A, Rasche V (2008). Local excitation black blood imaging at 3T: application to the carotid artery wall. Magn Reson Med.

[17] Bornstedt A, Burgmaier M, Hombach V, Marx N, Rasche V (2009). Dual stack black blood carotid artery CMR at 3T: application to wall thickness visualization. J Cardiovasc Magn Reson.

[18] Zhang S, Cai J, Luo Y, Han C, Polissar NL, Hatsukami TS, Yuan C (2003). Measurement of carotid wall volume and maximum area with contrast-enhanced 3D MR imaging: initial observations. Radiology.

[19] Fan Z, Zhang Z, Chung YC, Weale P, Zuehlsdorff S, Carr J, Li D (2010). Carotid arterial wall MRI at 3T using 3D variable-flip-angle turbo spin-echo (TSE) with flow-sensitive dephasing (FSD). J Magn Reson Imaging.

[20] Brittain JH, Hu BS, Wright GA, Meyer CH, Macovski A, Nishimura DG (1995). Coronary angiography with magnetization-prepared T2 contrast. Magn Reson Med.

[21] Giri S, Chung YC, Merchant A, Mihai G, Rajagopalan S, Raman SV, Simonetti OP (2009). T2 quantification for improved detection of myocardial edema. J Cardiovasc Magn Reson.

[22] Shea SM, Deshpande VS, Chung YC, Li D (2002). Three-dimensional true-FISP imaging of the coronary arteries: improved contrast with T2-preparation. J Magn Reson Imaging.

[23] Koktzoglou I, Li D (2007). Submillimeter isotropic resolution carotid wall MRI with swallowing compensation: imaging results and semiautomated wall morphometry. J Magn Reson Imaging.

